# Inextendibility of spacetimes and Lorentzian length spaces

**DOI:** 10.1007/s10455-018-9637-x

**Published:** 2018-11-10

**Authors:** James D. E. Grant, Michael Kunzinger, Clemens Sämann

**Affiliations:** 1 Department of Mathematics, University of Surrey, Guildford, UK; 2 Faculty of Mathematics, University of Vienna, Vienna, Austria

**Keywords:** Length spaces, Lorentzian length spaces, Causality theory, Synthetic curvature bounds, Triangle comparison, Metric geometry, Inextendibility, 53C23, 53C50, 53B30, 53C80, 83C75

## Abstract

We study the low-regularity (in-)extendibility of spacetimes within the synthetic-geometric framework of Lorentzian length spaces developed in Kunzinger and Sämann (Ann Glob Anal Geom 54(3):399–447, 2018). To this end, we introduce appropriate notions of geodesics and timelike geodesic completeness and prove a general inextendibility result. Our results shed new light on recent analytic work in this direction and, for the first time, relate low-regularity inextendibility to (synthetic) curvature blow-up.

## Introduction

One can distinguish between two main lines of research in low-regularity geometry. One approach is analytical, where one lowers the differentiability assumptions on, for example, (pseudo-)Riemannian metrics below the level where curvature can be classically defined. For example, one can study geometrical properties of (pseudo-)Riemannian metrics that have regularity $C^0$, $C^{0, \alpha }$ or $C^{1, 1}$, etc., or so-called Geroch–Traschen metrics, for which the Christoffel symbols are $L^2_{\mathrm {loc}}$, and the curvature is well-defined as a distribution [16, 28, 39]. The other approach to studying low-regularity geometries is by “synthetic” or metric space methods. Here, curvature bounds for Alexandrov spaces and CAT(*k*) spaces are defined in terms of comparison properties of geodesic triangles.

In the context of low-regularity Riemannian geometry, examples of a result of an analytical nature would be DeTurck and Kazdan’s study concerning harmonic coordinates [9], Taylor’s results on regularity of isometries [42] and Lytchak and Yaman’s result [30] that minimising curves for $C^{0, \alpha }$ Riemannian manifolds are $C^{1, \beta }$ curves, where $\beta = \frac{\alpha }{2-\alpha }$. Examples in this direction in the Lorentzian setting are the positive mass theorem for distributional curvature [19, 27], work on cone structures [6, 11, 32] and the recent work of extending the classical singularity theorems to $\mathcal {C}^{1,1}$-regularity [17, 25, 26], which in turn builds on previous results in low-regularity Lorentzian geometry and causality [7, 23, 24, 31, 37].

In the synthetic direction, the theory of Alexandrov spaces with curvature bounded above and/or below is well-developed as an appropriate generalisation of Riemannian geometry with sectional curvature bounds (see, for instance, [2, 4, 36]), and the work of Lott–Villani–Sturm gives a generalisation of the notion of a Riemannian metric with lower bound on the Ricci curvature to metric measure spaces [29, 40, 41].

In this paper, we will concentrate on a generalisation of Lorentzian geometry suitable for the low-regularity setting. More precisely, we shall be interested in the problem of finding low-regularity extensions of spacetimes. Concerning this question, approached from the analytical side, several fundamental contributions have appeared recently. Of particular relevance to us, Sbierski has shown the $C^0$-inextendibility of the Schwarzschild solution [38]. Building upon Sbierski’s work, Galloway, Ling and Sbierski established that global hyperbolicity combined with timelike geodesic completeness implies $C^0$-inextendibility. Further developments in this direction are due to Galloway–Ling and Graf–Ling (see below). In a related direction, Dafermos and Luk have recently shown $C^0$-extendibility of the interior of the Kerr solution [10].

In this paper, we will concentrate on the synthetic-geometric approach to extendibility. In [22], the theory of Lorentzian length spaces has been developed, which will form the framework of the present work. In this more axiomatic approach, there is a notion of a geodesic (as a locally length-maximising curve), which is not available in the more analytical direction of research. Therefore, it is possible to mimic the classical proof that geodesic completeness implies inextendibility (see, for example, [3, Prop. 6.16]). Moreover, within this picture, it becomes clear precisely what minimal geometric properties are underlying certain analytical extension results. In particular, for the first time, our approach allows us to directly relate low-regularity inextendibility with (synthetic) curvature blow-up. Such a result does not appear to be feasible in a purely analytical approach, due to the lack of a notion of a curvature for the extended spacetime.

An additional advantage of our synthetic approach is that there is no requirement for the introduction of coordinate systems, and regularity conditions (such as existence of smooth structures, or a certain level of differentiability) never arise. In this regard, it should perhaps be noted that in the analytical work on low-regularity extensions, one has to carry out standard geometrical constructions on the original manifold. As such, even though one works in a coordinate chart of the extended manifold in which the metric is merely continuous, the metric on the intersection of the original manifold with the coordinate chart must be $C^2$-regular.^1^ One could compare this situation with, for example, the fact that the Nash–Kuiper theorem [21, 34] implies that the flat metric on $T^2$ can be induced from a $C^1$ map $T^2 \rightarrow \mathbb {R}^3$.^2^ In the coordinate system in which the map is $C^1$, the induced metric will be merely $C^0$, even though we know that there exists a coordinate system in which the metric is smooth. As such, one could consider a more general notion of $C^0$ extensions of spacetimes, where one allows the regularity of the metric on the original manifold to drop. On the contrary, in our approach, such issues never arise. In fact, the extensions that we consider need not even be manifolds.

Our main references for Lorentzian geometry and causality theory are [3, 8, 33, 35], as well as [7] for the case of continuous Lorentzian metrics.

The plan of the paper is as follows: In Sect. 2 we briefly recall some main concepts and results on Lorentzian length spaces. Section 3 introduces extensions of Lorentzian (pre)length spaces, relates them to extensions of spacetimes and shows that the future or past boundary of an extension is non-empty. In Sect. 4 we define geodesics in the synthetic setting and show that this notion reduces precisely to that of pregeodesics for spacetimes. We also demonstrate that, as in the smooth case, extendibility as a geodesic is equivalent to continuous extendibility. In Sect. 5 we define an analogue of timelike completeness: a Lorentzian pre-length space is said to have property (TC) if all inextendible timelike geodesics have infinite length. This is the key property on which our main inextendibility result (Theorem 5.3) rests. We then establish connections between inextendibility and the occurrence of synthetic causal curvature singularities. Finally, in Sect. 6 we relate the results of the present work to the recent advances in the study of the low-regularity inextendibility of spacetimes.

## A short introduction to Lorentzian length spaces

Here we briefly recall some basic notions and results from the theory of Lorentzian length spaces, following [22], to which we refer for further details and proofs.

A set *X* endowed with a preorder $\le$ and a transitive relation $\ll$ contained in $\le$ is called a *causal space*. We write $x<y$ if $x\le y$ and $x\ne y$. If $x\ll y$, respectively, $x\le y$ we call *x* and *y* timelike, respectively, causally related. Chronological and causal futures and pasts $I^\pm (x)$, $J^\pm (x)$ of a point *x* are then defined in the usual manner based on these relations.

If *X* is, in addition, equipped with a metric *d* and a lower semicontinuous map $\tau :X\times X \rightarrow [0, \infty ]$ that satisfies the reverse triangle inequality $\tau (x,z)\ge \tau (x,y) + \tau (y,z)$ (for all $x\le y\le z$), as well as $\tau (x,y)=0$ if $x\nleq y$ and $\tau (x,y)>0 \Leftrightarrow x\ll y$, then $(X,d,\ll ,\le ,\tau )$ is called a *Lorentzian pre-length space* and $\tau$ is called the *time separation function* of *X*. Note that lower semicontinuity of $\tau$ implies that $I^{\pm }(x)$ is open, for any $x \in X$.

A non-constant curve $\gamma :I\rightarrow X$ (*I* an interval) is called (future-directed) *causal (timelike)* if $\gamma$ is locally Lipschitz continuous and if for all $t_1,t_2\in I$ with $t_1<t_2$ we have $\gamma (t_1)\le \gamma (t_2)$ ($\gamma (t_1)\ll \gamma (t_2)$). It is called *null* if, in addition to being causal, no two points on the curve are related with respect to $\ll$. For strongly causal continuous Lorentzian metrics, this notion of causality coincides with the usual one [22, Prop. 5.9]. In analogy to the theory of metric length spaces, the length of a causal curve is defined via the time separation function: For $\gamma :[a,b]\rightarrow X$ future-directed causal, we set

$$\begin{aligned} L_\tau (\gamma ):= \inf \left\{ \sum _{i=0}^{N-1} \tau (\gamma (t_i),\gamma (t_{i+1})): a=t_0<t_1<\ldots <t_N=b,\ N\in \mathbb {N}\right\} . \ \end{aligned}$$

If the interval is (half-)open, say $$I=[a,b)$$, then the infimum is taken over all partitions with $$a=t_0<t_1<\ldots<t_N<b$$, and similarly for the other cases. For smooth and strongly causal spacetimes (*M*, *g*), this notion of length coincides with the usual one: $$L_\tau (\gamma )=L_g(\gamma )$$ [22, Prop. 2.32]. A future-directed causal curve $$\gamma :[a,b]\rightarrow X$$ is *maximal* if it realises the time separation, i.e. if $$L_\tau (\gamma ) = \tau (\gamma (a),\gamma (b))$$.

Standard causality conditions (chronology, (strong) causality, global hyperbolicity, etc.) can also be imposed on Lorentzian pre-length spaces, and substantial parts of the causal ladder [33] continue to hold in this general setting. A Lorentzian pre-length space *X* is called *causally path-connected* if for all $x,y\in X$ with $x\ll y$ (respectively, $x<y$) there is a future-directed timelike (respectively, causal) curve from *x* to *y*. A neighbourhood *U* of *x* is called *causally closed* if the relation $\le$ is closed in $\bar{U}\times \bar{U}$, and *X* itself is called *locally causally closed* if every point has a causally closed neighbourhood.

A key technical tool in smooth semi-Riemannian geometry is the existence of convex neighbourhoods, in which the causality is particularly simple and where one has a complete description of length-maximising curves. The analogue of this notion in the present context is the following: A Lorentzian pre-length space *X* is called *localisable* if any $x\in X$ has an open, so-called *localising* neighbourhood $\Omega _x$ such that:

(i) The *d*-length of all causal curves contained in $\Omega _x$ is uniformly bounded.
(ii) There is a continuous map $$\omega _x:\Omega _x \times \Omega _x\rightarrow [0,\infty )$$ such that $$(\Omega _x, d|_{\Omega _x\times \Omega _x},$$$$\ll |_{\Omega _x\times \Omega _x},\le |_{\Omega _x\times \Omega _x}, \omega _x)$$ is a Lorentzian pre-length space, and for every $$y\in \Omega _x$$ we have $$I^\pm (y)\cap \Omega _x\ne \emptyset $$.
(iii) For all $p,q\in \Omega _x$ with $p<q$ there is a future-directed causal curve $\gamma _{p,q}$ from *p* to *q* that is maximal in $\Omega _x$ and satisfies $L_\tau (\gamma _{p,q}) = \omega _x(p,q) \le \tau (p,q)$.
  If, in addition, the neighbourhoods $\Omega _x$ can be chosen such that
(iv) Whenever $p,q\in \Omega _x$ satisfy $p\ll q$ then $\gamma _{p,q}$ is timelike and strictly longer than any future-directed causal curve in $\Omega _x$ from *p* to *q* that contains a null segment,

then $(X,d,\ll ,\le ,\tau )$ is called *regularly localisable*.

Lorentzian length spaces are close analogues of metric length spaces in the sense that the time separation function can be calculated from the length of causal curves connecting causally related points. Precisely, a locally causally closed, causally path-connected and localisable Lorentzian pre-length space is called a *Lorentzian length space* if $\tau = \mathcal {T}$, where for any $x,y\in X$ we set

$$\begin{aligned} \mathcal {T}(x,y):= \sup \{L_\tau (\gamma ):\gamma \text { future-directed causal from }x \text { to } y\}\,, \end{aligned}$$

if the set of future-directed causal curves from *x* to *y* is not empty. Otherwise let $\mathcal {T}(x,y):=0$. If, in addition, *X* is regularly localisable, then it is called a regular Lorentzian length space.

Any smooth strongly causal spacetime is an example of a regular Lorentzian length space (with metric $d = d^h$ induced by any Riemannian metric *h* on the spacetime). More generally, any spacetime with a continuous, strongly causal and causally plain metric (see the remark preceding Corollary 5.5) is a (strongly) localisable Lorentzian length space. Further examples are provided by certain Lorentz–Finsler spaces in the sense of [32] or, for the non-manifold setting, causal Fermion systems [12, 13].

The final concept from the theory of Lorentzian length spaces we are going to require below is that of synthetic curvature bounds, based on triangle comparison. We will confine ourselves to causal triangle comparison here, as this is the only one we are going to employ. By an *admissible causal geodesic triangle*, we mean a triple $(x,y,z)\in X^3$ with $x\ll y \le z$ or $x \le y \ll z$ such that $\tau (x,z)<\infty$ and such that the sides (if non-trivial) are realised by future-directed causal curves. Curvature bounds are formulated by comparing such triangles with triangles of the same side lengths in one of the Lorentzian model spaces $M_K$ of constant sectional curvature. Here,

1 $$\begin{aligned} M_K = \left\{ \begin{array}{ll} \tilde{S}^2_1(r) &{}\quad K=\frac{1}{r^2}\\ \mathbb {R}^2_1 &{}\quad K=0\\ \tilde{H}^2_1(r) &{}\quad K= -\frac{1}{r^2}. \end{array} \right. \end{aligned}$$

where $\tilde{S}^2_1(r)$ is the simply connected covering manifold of the two-dimensional Lorentzian pseudosphere $S^2_1(r)$ (i.e. de-Sitter space), $\mathbb {R}^2_1$ is two-dimensional Minkowski space, and $\tilde{H}^2_1(r)$ is the simply connected covering manifold of the two-dimensional Lorentzian pseudohyperbolic space (i.e. anti-de-Sitter space) . In order to guarantee the existence of comparison triangles in one of the model spaces, one needs to impose size restrictions of the following kind: Given $K\in \mathbb {R}$, let $(a,b,c)\in \mathbb {R}_+^3$ with $c\ge a+b$. If $c=a+b$, then let $c<\frac{\pi }{\sqrt{K}}$. (Here, $\frac{\pi }{\sqrt{K}}:=\infty$ if $K\le 0$). Otherwise, if $K<0$ then assume $c<\frac{\pi }{\sqrt{-K}}$. Then (*a*, *b*, *c*) is said to *satisfy timelike size bounds* for *K*. These bounds ensure the existence of comparison triangles in the corresponding model space.

Using this terminology, a Lorentzian pre-length space $(X,d,\ll ,\le ,\tau )$ is said to have causal curvature bounded below (above) by $K\in \mathbb {R}$ if every point in *X* has a neighbourhood *U* such that:

(i) $\tau |_{U\times U}$ is finite and continuous.
(ii) Whenever $x,y \in U$ with $x < y$, there exists a causal curve $\alpha$ in *U* with $L_\tau (\alpha ) = \tau (x,y)$.
(iii) If (*x*, *y*, *z*) is an admissible causal geodesic triangle in *U*, realised by maximal causal curves (or a constant curve, respectively) $\alpha , \beta , \gamma$ whose side lengths satisfy timelike size bounds for *K*, and if $(\bar{x},\bar{y},\bar{z})$ is a comparison triangle of (*x*, *y*, *z*) in $M_K$ realised by causal geodesics (or a constant curve) $\bar{\alpha }$, $\bar{\beta }$, $\bar{\gamma }$, then whenever *p*, *q* are points on the timelike sides of (*x*, *y*, *z*) and $\bar{p}$, $\bar{q}$ are corresponding points of the timelike sides of $(\bar{x},\bar{y},\bar{z})$, we have $\tau (p,q)\le \bar{\tau }(\bar{p}, \bar{q})$ (respectively, $\tau (p,q)\ge \bar{\tau }(\bar{p}, \bar{q}))$.

Such a neighbourhood *U* is called a *comparison neighbourhood with respect to*$M_K$.

## Extensions

We start the main part of our work by defining the notion of an *extension* of a Lorentzian pre-length space, requiring only conditions that are natural within our setting. This concept is fully compatible with the usual notion of extension for spacetimes, see Proposition 3.5.

### Definition 3.1

Let $(X,d,\ll ,\le ,\tau )$ be a Lorentzian pre-length space. A Lorentzian pre-length space $(\tilde{X},\tilde{d},\tilde{\ll },\tilde{\le },\tilde{\tau })$ is called an *extension* of $(X,d,\ll ,\le ,\tau )$ if

(i) the metric space $(\tilde{X}, \tilde{d})$ is connected,
(ii) there exists an isometry $\iota :(X,d)\rightarrow (\tilde{X}, \tilde{d})$ of metric spaces,
(iii) the image $\iota (X)$ is a proper, open subset of $\tilde{X}$,
(iv) $\iota$ preserves $\ll$ and $\le$, i.e. $\forall x,y\in X$: if $x\le y$ then $\iota (x)\ \tilde{\le }\ \iota (y)$ and if $x\ll y$ then $\iota (x)\ \tilde{\ll }\ \iota (y)$, and
(v) a curve $\gamma :I \rightarrow X$ is timelike (respectively, causal) if and only if $\iota \circ \gamma$ is timelike (respectively, causal) in $(\tilde{X},\tilde{d},\tilde{\ll },\tilde{\le },\tilde{\tau })$. Furthermore, $\iota$ preserves $\tau$-lengths, i.e. for any $\le$-causal curve $\gamma :I\rightarrow X$ we have
  2 $$\begin{aligned} L_\tau (\gamma ) = L_{\tilde{\tau }}(\iota \circ \gamma )\,. \end{aligned}$$

In this case $(X,d,\ll ,\le ,\tau )$ is called *extendible*. If no extension exists, then $(X,d,\ll ,\le ,\tau )$ is called *inextendible* (as a Lorentzian pre-length space).

### Remark 3.2

Of course, this definition also applies to Lorentzian length spaces, i.e. a Lorentzian length space is *extendible* if there is a Lorentzian length space $(\tilde{X},\tilde{d},\tilde{\ll },\tilde{\le },\tilde{\tau })$ and $\iota :(X,d)\rightarrow (\tilde{X}, \tilde{d})$ with the above properties 3.1-(v). In this case conditions (iv) and (v) slightly simplify.

### Lemma 3.3

Let $(\tilde{X},\tilde{d},\tilde{\ll },\tilde{\le },\tilde{\tau })$ be an extension of $(X,d,\ll ,\le ,\tau )$, where both are Lorentzian length spaces. Then $\tilde{\tau } \circ (\iota \times \iota ) \ge \tau$.

### Proof

Let $p,q\in X$ with $\tau (p,q)>0$ (if $\tau (p,q)=0$ there is nothing to do). Let $\gamma$ be a future-directed $\le$-causal curve from *p* to *q* (which exists due to $p\le q$ and the causal path-connectedness of *X*). Then $\iota \circ \gamma$ is $\tilde{\le }$-causal and $L_\tau (\gamma )=L_{\tilde{\tau }}(\iota \circ \gamma )\le \tilde{\mathcal {T}}(\iota (p),\iota (q)) = \tilde{\tau }(\iota (p),\iota (q))$. Taking the supremum over all future-directed $\le$-causal curves from *p* to *q*, we get $\mathcal {T}(p,q)\le \tilde{\tau }(\iota (p),\iota (q))$ and since $\mathcal {T}=\tau$ the claim follows. $\square$

The following lemma shows that condition (v) of Definition 3.1 required of an extension is in fact not too strong. Moreover, it demonstrates that for smooth strongly causal spacetimes the time separation function determines the metric completely.

### Lemma 3.4

Let (*M*, *g*) and $(\tilde{M},\tilde{g})$ be smooth spacetimes (of the same dimension) with time separation functions $\tau$ and $\tilde{\tau }$, respectively. Let (*M*, *g*) be strongly causal and let $\iota :M\rightarrow \tilde{M}$ be onto. Then $\iota$ is an isometry if and only if $\iota$ preserves causal curves and their lengths, i.e. a curve $\gamma$ is causal in *M* if and only if $\iota \circ \gamma$ is causal in $\tilde{M}$ and for such curves, $L_g(\gamma ) = L_{\tilde{g}}(\iota \circ \gamma )$.

### Proof

It is a classical result that goes back to Hawking, King and McCarthy [20] (cf. [33, Prop. 3.34] or [3, Thm. 4.17]) that $\iota$ is an isometry if and only if it preserves $\tau$. By definition of the time separation functions in spacetimes, this latter condition is, in turn, implied by $\iota$ preserving the *g*-lengths of causal curves. $\square$

Furthermore, in the case of spacetimes the above result implies that there is no difference between an extension in our sense, and in the usual sense of an isometric embedding (cf. [38, Def. 2.15]. To be precise, we have the following result:

### Proposition 3.5

Let (*M*, *g*) and $(\tilde{M}, \tilde{g})$ be smooth, strongly causal spacetimes (of the same dimension) and let $\iota :M\rightarrow \tilde{M}$ be a map such that $\iota (M)\subset \tilde{M}$. Then the induced Lorentzian length space of $(\tilde{M}, \tilde{g})$ extends the one coming from (*M*, *g*) via $\iota$ if and only if $\iota$ is a (smooth) isometric embedding.

### Proof

We start with the following observation: Let $\tilde{h}$ be any Riemannian metric on $\tilde{M}$ with induced metric $d^{\tilde{h}}$. This fixes the induced Lorentzian length space in the following sense: Any other Riemannian metric on $\tilde{M}$ also induces the manifold topology and the notion of locally Lipschitz continuous curves is preserved (cf. [8, Prop. 2.3.1]), thus fixing the spacetime $(\tilde{M}, \tilde{g})$ and any Riemannian background metric determines the resulting Lorentzian length space.

Assume that $(\tilde{M},d^{\tilde{h}},\tilde{\ll },\tilde{\le },\tilde{\tau })$ extends $(M,d^h,\ll ,\le ,\tau )$ via $\iota$. As $\iota (M)$ is an open and connected subset of $\tilde{M}$, we consider the spacetime $(\hat{M},\hat{g}):= (\iota (M),\tilde{g}|_{\iota (M)})$ with its time separation function $\hat{\tau }$. This means that

$$\begin{aligned} \hat{\tau }(\tilde{p},\tilde{q}) = \sup \left\{ L_{\tilde{g}}(\tilde{\gamma }): \tilde{\gamma } \text { f.d. causal curve from } \tilde{p}\text { to }\tilde{q} \text { with } \text {image}(\tilde{\gamma })\subseteq \iota (M)\right\} \,. \end{aligned}$$

By Definition 3.1,(v) a curve $\gamma :I\rightarrow M$ is causal if and only if $\iota \circ \gamma :I \rightarrow \hat{M}$ is causal in $(\hat{M},\hat{g})$. This together with (2) and [22, Prop. 2.32] implies that $\iota$ preserves $\hat{\tau }$, i.e.

$$\begin{aligned} \tau (p,q)=\hat{\tau }(\iota (p),\iota (q))\quad \forall p,q\in M\,. \end{aligned}$$

Thus, by [33, Prop. 3.34] $\iota$ is an isometry $(M,g)\rightarrow (\hat{M},\hat{g})$.

For the converse assume that $\iota$ is a smooth isometric embedding. Then we check points 3.1-(v) of Definition 3.1. As $\tilde{M}$ is connected by assumption, the first point follows. Pulling back $\tilde{h}$ to *M* gives a Riemannian metric $h:=\iota ^*(\tilde{h}|_{\iota (M)})$. Denoting its induced metric by $d^h$, we obtain a metric isometry $\iota :(M,d^h)\rightarrow (\tilde{M}, d^{\tilde{h}})$ and $\iota (M)$ is open and proper—giving the second and third point. Let $p,q\in M$ with $p<q$, i.e. there exists a future-directed causal curve $\gamma$ from *p* to *q*. As $\iota$ is an isometry of (*M*, *g*) and $(\tilde{M}, \tilde{g})$, the curve $\iota \circ \gamma$ is future-directed causal and connects $\iota (p)$ with $\iota (q)$. Thus $\iota (p)\tilde{<}\iota (q)$. The case for $p\ll q$ is completely analogous, giving the fourth point. Finally, let $\gamma :I\rightarrow M$ be a (locally Lipschitz continuous) curve. Then $\gamma$ is *g*-timelike/causal if and only if $\iota \circ \gamma$ is $\tilde{g}$-timelike/causal by the isometric embedding property. Moreover, by [22, Prop. 2.32] we have

$$\begin{aligned} L_\tau (\gamma ) = L_g(\gamma ) = L_{\tilde{g}}(\iota \circ \gamma ) = L_{\tilde{\tau }}(\iota \circ \gamma )\,. \end{aligned}$$

This gives the fifth point and finishes the proof. $\square$

To illustrate that one can have extensions that are not manifolds, we consider the following example, which is a Lorentzian version of [2, Ex. 4.2.5].

### Example 3.6

**Fig. 1:**
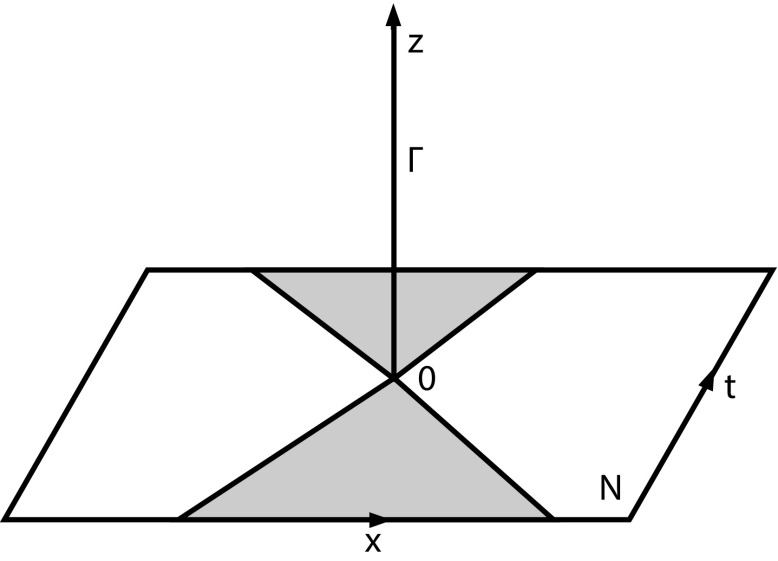
Non-manifold extension

Let $\mathbb {R}^2_1$ be two-dimensional Minkowski space and embed it into $\mathbb {R}^3$ as a plane through the origin orthogonal to the *z*-direction, i.e. $N:=\{(t,x,0): (t,x)\in \mathbb {R}^2\}$. We now add a half-ray to the origin and give the resulting space the structure of a Lorentzian length space. Let $\Gamma :=\{(0,0,z):z\ge 0\}$ and set $\tilde{M}:= N \cup \Gamma$ (Fig. 1). On *N* we use the relations from Minkowski space and on $\Gamma$ we define $Z_1:=(0,0,z_1)\ll Z_2:=(0,0, z_2)$ if $z_1 < z_2$, and $Z_1\le Z_2$ if $Z_1\ll Z_2$ or $Z_1=Z_2$. For $p=(t,x,0)\in N$ and $Z\in \Gamma$ we define $p\ll Z$ if $(t,x)\ll 0$ in $\mathbb {R}^2_1$ and analogously for the causal relation. We define the time separation function $\tau$ as the time separation function coming from Minkowski space on *N*, for points on $\Gamma$ we set $\tau ((0,0,z_1),(0,0,z_2)):=z_2-z_1$ if $z_1\le z_2$ (zero otherwise) and for $p=(t,x,0)\in N$ and $Z=(0,0,z)$ we set $\tau (p,Z):=\sqrt{t^2-x^2} + z$ if $p\le Z$ (and zero otherwise). As $\tau$ is continuous this gives a Lorentzian pre-length space. In fact, this construction gives a Lorentzian length space as it is clearly path-connected and locally causally closed. Moreover, it is regularly localisable since maximal causal curves always exist (they are the, possibly broken, straight lines) and the induced length agrees with the $\tau$-length by construction. Furthermore, it is not hard to see that $\tilde{M}$ is strongly causal. In this space maximal curves branch: every maximal curve from $J^-(0)$ to $J^+(0)$ has 0 as a branching point, as the curve is allowed to continue into *N* or $\Gamma$. This implies via [22, Cor. 4.13] that $\tilde{M}$ has timelike curvature unbounded below, i.e. a curvature singularity in the sense of [22, Def. 4.20]. Finally, $\tilde{M}$ extends $M\backslash \{(0,0)\}$, thereby providing an example of a non-manifold extension. Note that $\tilde{M}$ does not extend *M* since *M* is not embedded into $\tilde{M}$ as an open subset.

At this point we can introduce the past and future boundary of Lorentzian pre-length spaces with respect to an extension in complete analogy to the case of spacetime extensions, see [14, Def. 2.1].

### Definition 3.7

Let $(\tilde{X},\tilde{d},\tilde{\ll },\tilde{\le },\tilde{\tau })$ be a Lorentzian pre-length space extending the Lorentzian pre-length space $(X,d,\ll ,\le ,\tau )$ via the embedding $\iota$. The *future/past boundary*$\partial ^+(X)$ / $\partial ^-(X)$ of *X* is defined as the set of all points $\tilde{p}\in \partial \iota (X)$ that can be reached by a future-/past-directed $\tilde{\ll }$-timelike curve $\gamma :[0,1]\rightarrow \tilde{X}$ such that $\gamma ([0,1))\subseteq \iota (X)$ and $\gamma (1)=\tilde{p}$.

The following result establishes that for any extension of a Lorentzian length space the future or past boundary is non-empty. It is a direct analogue of [38, Lemma 2.17].

### Lemma 3.8

Let $(\tilde{X},\tilde{d},\tilde{\ll },\tilde{\le },\tilde{\tau })$ be an extension of $(X,d,\ll ,\le ,\tau )$, where both are Lorentzian length spaces, and denote the corresponding isometry by $\iota$. Then there is a $\tilde{\ll }$-timelike curve $\tilde{\gamma }:[0,1]\rightarrow \tilde{X}$ such that $\tilde{\gamma }([0,1))\subseteq \iota (X)$ and $\tilde{\gamma }(1)\in \tilde{X}\backslash \iota (X)$, i.e. $\partial ^+(X)\cup \partial ^-(X)\ne \emptyset$.

### Proof

Since $\iota (X)$ is a proper and open subset of $\tilde{X}$ and $\tilde{X}$ is connected, we get that $\partial \iota (X)\ne \emptyset$. Let $\tilde{p}\in \partial \iota (X)$ and let $\tilde{\Omega }$ be a localising neighbourhood of $\tilde{p}$ in $\tilde{X}$. Then, $\tilde{I}^\pm (\tilde{p})\cap \tilde{\Omega }\ne \emptyset$ and let $\tilde{q}\in \tilde{I}^-(\tilde{p})\cap \tilde{\Omega }$. We now consider two cases. First, if $\tilde{q}\in \iota (X)$, then since $\tilde{q}\tilde{\ll }\tilde{p}$ there is a $\tilde{\ll }$-timelike curve $\tilde{\gamma }:[0,1]\rightarrow \tilde{X}$ such that $\tilde{\gamma }(0)=\tilde{q}$, $\tilde{\gamma }(1)=\tilde{p}$. Set $s_0:=\sup \{s\in [0,1]: \tilde{\gamma }([0,s])\subseteq \iota (X)\}$, then since $\iota (X)$ is open and $\tilde{p}\in \partial \iota (X)$ we have $\tilde{\gamma }(s_0)\in \tilde{X}\backslash \iota (X)$. Reparametrising $\tilde{\gamma }|_{[0,s_0]}$ to [0, 1] yields the result. The second case is when $\tilde{q}\in \tilde{X}\backslash \iota (X)$. Now $\tilde{I}^+(\tilde{q})\cap \tilde{\Omega }$ is a neighbourhood of $\tilde{p}\in \partial \iota (X)$, thus $\iota (X)\cap (\tilde{I}^+(\tilde{q})\cap \tilde{\Omega })\ne \emptyset$. Let $\tilde{r}\in \iota (X)\cap (\tilde{I}^+(\tilde{q})\cap \tilde{\Omega })$, then $\tilde{q}\tilde{\ll }\tilde{r}$ and the result follows as in the first case by arguing into the past. $\square$

## Geodesics

In this synthetic approach we have the tools at hand to define causal geodesics as locally length-maximising curves. Furthermore, we establish that for spacetimes the synthetic notion is compatible with the analytical one.

### Definition 4.1

Let $(X,d,\ll ,\le ,\tau )$ be a localising Lorentzian pre-length space and let $\gamma :I\rightarrow X$ be a future-directed causal curve. Then $\gamma$ is a *geodesic* if for every $t_0\in I$ there exists a localising neighbourhood $\Omega$ of $\gamma (t_0)$ and a neighbourhood $J=[c,d]$ of $t_0$ in *I* such that $\gamma |_{J}$ is maximal in $\Omega$ from $\gamma (c)$ to $\gamma (d)$.

### Remark 4.2

Let $\gamma :I\rightarrow X$ be a geodesic and let $t_0\in I$, and $\Omega$ a localising neighbourhood of $\gamma (t_0)$ as above. Then

$$\begin{aligned} L_\tau (\gamma |_{[c,d]}) = \omega _{\gamma (t_0)}(\gamma (c),\gamma (d))\,, \end{aligned}$$

where $\omega _{\gamma (t_0)}$ is the local time separation function on $\Omega$, cf. [22, Def. 3.16].

To show that for a smooth and strongly causal spacetime this notion is equivalent to the notion of causal pregeodesics, we need the following lemma stating a general property of strongly causal Lorentzian length spaces.

### Lemma 4.3

Let $(X,d,\ll ,\le ,\tau )$ be a strongly causal Lorentzian length space. Then for all $x\in X$ and every localising neighbourhood $\Omega$ of *x* with local time separation function $\omega$ there is a neighbourhood *U* of *x*, $U\subseteq \Omega$ such that $\omega |_{U\times U}$ is completely determined by $\tau$: $\forall p,q\in U:\ \omega (p,q)=\tau (p,q)$. In particular, $\tau$ is continuous on a neighbourhood of the diagonal in $X\times X$.

### Proof

Let $x\in X$ and let $\Omega$ be a localising neighbourhood of *x* with local time separation function $\omega$. By strong causality and [22, Lemma 2.38(iii)] there is a neighbourhood *U* of *x* with $U\subseteq \Omega$ such that all causal curves with endpoints in *U* are contained in $\Omega$. Let $p,q\in U$ with $p<q$, then by the properties of $\Omega$ (see Sect. 2) there is a causal curve $\gamma _{pq}$ that is maximal in $\Omega$ from *p* to *q* with $L_\tau (\gamma _{pq}) = \omega (p,q)$. As $p,q\in U$, any causal curve connecting these points is contained in $\Omega$. Thus, $\gamma _{pq}$ is maximal even in *X*, and consequently, we have $\tau (p,q) = \mathcal {T}(p,q) = L_\tau (\gamma _{pq}) = \omega (p,q)$. The neighbourhood of the diagonal can be chosen to be the union of all such $U\times U$ as above. $\square$

With the above lemma we can now establish the promised compatibility.

### Theorem 4.4

Let (*M*, *g*) be a smooth, strongly causal spacetime and let $(M,d^h,\ll ,\le ,\tau )$ be the induced Lorentzian length space [22, Ex. 3.24(i)]. Then a causal pregeodesic of (*M*, *g*) is a geodesic in the sense of Definition 4.1 and vice versa.

### Proof

First, let $\gamma :I\rightarrow M$ be a causal pregeodesic of (*M*, *g*), which we can assume without loss of generality to be already parametrised as a geodesic. The localising neighbourhoods can be chosen to be (totally) normal neighbourhoods. Let $t_0\in I$ and let *U* be a totally normal neighbourhood of $\gamma (t_0)$. Let $J=[c,d]$ be a neighbourhood of $t_0$ in *I* such that $\gamma (J)\subseteq U$ and set $x:=\gamma (c), y:=\gamma (d)$. Since $\gamma$ is a geodesic, it has to be the radial geodesic from *x* to *y* in *U*. As such it is maximal in *U* and because $L_g=L_\tau$ by [22, Prop. 2.32] we obtain

$$\begin{aligned} L_\tau (\gamma |_{[c,d]}) = L_g(\gamma |_{[c,d]}) = \sqrt{-g_x(\exp _x^{-1}(y),\exp _x^{-1}(y))}=\omega (x,y)\,. \end{aligned}$$

Conversely, let $\gamma :I\rightarrow M$ be a geodesic in the sense of Definition 4.1. As this is a local question, we can cover $\gamma (I)$ by open sets *U*, where $U\subseteq \Omega$ are as in the proof of Lemma 4.3, and show that the segment of $\gamma$ in any such *U* is a pregeodesic with respect to *g*. In fact, let $t_0\in I$ with $\gamma (t_0)\in U_0\subseteq \Omega _0$ and let $J\subseteq I$ be an interval around $t_0$ such that $\gamma (J)\subseteq U_0$. Let $s_1, s_2 \in J$ with $s_1<s_2$, then we get from Lemma 4.3 that

$$\begin{aligned} L_\tau (\gamma |_{[s_1,s_2]}) = \omega (\gamma (s_1),\gamma (s_2)) = \tau (\gamma (s_1),\gamma (s_2))\,. \end{aligned}$$

Therefore, again since $L_g=L_\tau$, $\gamma$ is maximal on $[s_1,s_2]$ and hence $\gamma$ is a pregeodesic (see, for example, [3, Thm. 4.13]). $\square$

Note that the above proof also shows that the property of being timelike agrees for causal pregeodesics of (*M*, *g*) and geodesics in the sense of Definition 4.1 (contrary to the case for arbitrary curves, cf. [22], Ex. 2.22]).

### Definition 4.5

Let $(X,d,\ll ,\le ,\tau )$ be a localising Lorentzian pre-length space and let $\gamma :[a,b)\rightarrow X$ be a future-directed geodesic. Then $\gamma$ is *extendible as a geodesic* if there exists a (future-directed) geodesic $\bar{\gamma }:[a,b]\rightarrow X$ with $\bar{\gamma }|_{[a,b)}=\gamma$. Otherwise, $\gamma$ is called *inextendible as a geodesic*.

A well-known property of geodesics in smooth semi-Riemannian manifolds is the fact that extendibility as a geodesic is equivalent to continuous extendibility. Its standard proof relies on the existence of convex neighbourhoods. The following result is an analogue in the setting of Lorentzian pre-length spaces, with localising neighbourhoods working as a substitute.

### Proposition 4.6

Let $(X,d,\ll ,\le ,\tau )$ be a strongly causal and localising Lorentzian pre-length space and let $\gamma :[a,b)\rightarrow X$ be a future-directed geodesic. Then $\gamma$ is extendible as a geodesic if and only if it is extendible as a continuous curve to [*a*, *b*].

### Proof

Only the ‘if’ part requires a proof, so let us suppose that $\gamma :[a,b]\rightarrow X$ is continuous and that $\gamma |_{[a,b)}$ is a geodesic. Let $\Omega$ be a localising neighbourhood of $\gamma (b)$ and choose $c\in (a,b)$ such that $\gamma ([c,b]) \subseteq \Omega$. Then for any $t\in (c,b)$ we have

$$\begin{aligned} L_\tau (\gamma |_{[c,t]}) = \omega (\gamma (c),\gamma (t)), \end{aligned}$$

where $\omega \equiv \omega _{\gamma (b)}$ is the local time separation function on $\Omega$. As $t\nearrow b$, the right hand side of this equation converges to $\omega (\gamma (c),\gamma (b))$. Concerning the left hand side, for any $n\in \mathbb {N}$ with $\frac{1}{n}<b-c$ denote by $\gamma _n :[c,b]\rightarrow X$ a linear reparametrisation of $\gamma |_{[c,b-\frac{1}{n}]}$. Then the $\gamma _n$ converge uniformly to $\gamma$ on [*c*, *b*]. Therefore, [22, Prop. 3.17] implies that

$$\begin{aligned} L_\tau (\gamma |_{[c,b]}) \ge \limsup _n L_\tau (\gamma _n) = \limsup _n \omega (\gamma (c),\gamma (b-1/n)) = \omega (\gamma (c),\gamma (b)). \end{aligned}$$

As the converse of this inequality holds by the definition of localisability (cf. Sect. 2), the claim follows. $\square$

## Timelike completeness and inextendibility

As discussed in the introduction, our approach allows us to mimic the proof from the smooth case that geodesic completeness implies inextendibility, i.e. [3, Prop. 6.16]. We first introduce an appropriate notion of timelike geodesic completeness for Lorentzian pre-length spaces.

### Definition 5.1

Let $(X,d,\ll ,\le ,\tau )$ be a localising Lorentzian pre-length space, then *X* is said to have property (*TC*) if all inextendible timelike geodesics have infinite $\tau$-length.

This notion is equivalent to timelike geodesic completeness in the case of smooth and strongly causal spacetimes:

### Lemma 5.2

Let $(M,d^h,\ll ,\le ,\tau )$ be the Lorentzian length space induced by a smooth and strongly causal spacetime (*M*, *g*). Then (*M*, *g*) is timelike geodesically complete if and only if $(M,d^h,\ll ,\le ,\tau )$ has property (*TC*).

### Proof

First, let (*M*, *g*) be not timelike geodesically complete, so that there exists an inextendible timelike geodesic (without loss of generality inextendible to the future) $\gamma :[a,b)\rightarrow M$, with $b<\infty$, thus $L_g(\gamma )<\infty$. Since $L_g=L_\tau$ by [22, Prop. 2.32], Theorem 4.4 implies that property (TC) cannot hold. Conversely, let (*M*, *g*) be timelike geodesically complete and let $\gamma :[0,b)\rightarrow M$ be an inextendible timelike geodesic (in the sense of Definition 4.1). Then by Theorem 4.4$\gamma$ is a timelike pregeodesic of (*M*, *g*), hence by completeness $L_g(\gamma )=\infty$ (cf. [35, p. 154]). Since $L_g = L_\tau$, property (*TC*) follows. $\square$

Property (TC) does guarantee inextendibility, as the following result shows.

### Theorem 5.3

Let $(X,d,\ll ,\le ,\tau )$ be a strongly causal Lorentzian length space that has property (*TC*). Then $(X,d,\ll ,\le ,\tau )$ is inextendible as a regular Lorentzian length space.

### Proof

Assume, to the contrary, that there exists a regular Lorentzian length space $(\tilde{X},\tilde{d},\tilde{\ll },\tilde{\le },\tilde{\tau })$ that extends $(X,d,\ll ,\le ,\tau )$. By Lemma 3.8 there is a (without loss of generality) future-directed $\tilde{\ll }$-timelike curve

$\tilde{\gamma }:[0,1]\rightarrow \tilde{X}$ with $\tilde{\gamma }([0,1))\subseteq \iota (X)$ and $\tilde{\gamma }(1)=\tilde{p}\in \tilde{X}{\setminus }\iota (X)$. Let $\tilde{U}$ be a localising neighbourhood of $\tilde{p}$ (with respect to $\tilde{X}$) and $\tilde{\omega }$ its local time separation function. Let $t_0\in [0,1)$ be such that $\tilde{\gamma }([t_0,1])\subseteq \tilde{U}$. Consequently, $q:=\tilde{\gamma }(t_0)\in \tilde{U} \cap \iota (X)$ and $q\tilde{\ll } \tilde{p}$. Thus, there is an—in $\tilde{U}$—$\tilde{\tau }$-maximal curve $\tilde{\gamma }_{q,\tilde{p}}:[0,1]\rightarrow \tilde{U}$ from *q* to $\tilde{p}$, which is $\tilde{\ll }$-timelike by regularity, see [22, Thm. 3.18]. Since $\iota (X)$ is open, $q\in \iota (X)$ and $\tilde{p}\notin \iota (X)$ there is a $t_*\in (0,1)$ such that $\tilde{\gamma }_{q,\tilde{p}}([0,t_*))\subseteq \iota (X)$ and $\tilde{r}:=\tilde{\gamma }_{q,\tilde{p}}(t_*)\notin \iota (X)$. Then $\tilde{\gamma }_{q,\tilde{p}}|_{[0,t_*)}:[0,t_*)\rightarrow \tilde{U}\cap \iota (X)$ and we set $\lambda :=\iota ^{-1}\circ \tilde{\gamma }_{q,\tilde{p}}|_{[0,t_*)}$. By Definition 3.1,(v), $\lambda$ is $\ll$-timelike. We claim that $\lambda$ is a timelike $\tau$-geodesic. To this end, recall that a maximal causal curve is maximal on any subinterval, see [22, Prop. 2.34,(ii)]. Fix any $0\le s_0 < t_*$, and let *V* be a neighbourhood of $\lambda (s_0)$ with $\iota (V)\subseteq \tilde{U}$. As *X* is strongly causal, there exists a neighbourhood $V'\subseteq V$ of $\lambda (s_0)$ such that any causal curve that starts and ends in $V'$ is contained in *V*. Now suppose that $s_1\le s_0<s_2$ are so close that $\lambda |_{[s_1,s_2]}$ is contained in $V'$. Then in particular any future-directed $\le$-causal curve connecting $\lambda (s_1)$ to $\lambda (s_2)$ remains entirely in *V*. By Definition 3.1,(v) we therefore obtain

$$\begin{aligned} \begin{aligned} L_\tau (\lambda |_{[s_1,s_2]})&= L_{\tilde{\tau }}(\iota \circ \lambda |_{[s_1,s_2]})\\&=\max \left\{ L_{\tilde{\tau }}(\tilde{\alpha }):\tilde{\alpha } \text { f.d. } {\tilde{\le }\text {-causal from }} \iota \circ \lambda (s_1)\text { to }\iota \circ \lambda (s_2) \text { in } \tilde{U} \right\} \\&\ge \max \left\{ L_{\tilde{\tau }}(\iota \circ \alpha ):\alpha \text { f.d. } {\le \text {-causal from }} \lambda (s_1) \text { to }\lambda (s_2)\text { in } V \right\} \\&=\max \left\{ L_{\tau }(\alpha ):\alpha \text { f.d. } {\le \text {-causal from }} \lambda (s_1) \text { to } \lambda (s_2) \text { in } V\right\} \\&=\max \left\{ L_{\tau }(\alpha ):\alpha \text { f.d. } {\le \text {-causal from }} \lambda (s_1) \text { to } \lambda (s_2) \text { in }X \right\} \\&= \mathcal {T}(\lambda (s_1),\lambda (s_2)))\ge L_\tau (\lambda |_{[s_1,s_2]})\,. \end{aligned} \end{aligned}$$

Thus, $L_\tau (\lambda |_{[s_1,s_2]}) = \mathcal {T}(\lambda (s_1),\lambda (s_2))) = \tau (\lambda (s_1),\lambda (s_2)))$. By Lemma 4.3, any local time separation function is completely determined by $\tau$ on $V'$; hence, the above shows that $\lambda$ is a geodesic in *X*. Moreover, the length of $\lambda$ is given by

$$\begin{aligned} L_\tau (\lambda ) = L_{\tilde{\tau }}(\iota \circ \lambda ) = \lim _{t\nearrow t_*}L_{\tilde{\tau }}(\tilde{\gamma }_{q,\tilde{p}}|_{[0,t]}) = \lim _{t\nearrow t_*}\tilde{\omega } (q,\tilde{\gamma }_{q,\tilde{p}}(t)) = \tilde{\omega }(q,r)<\infty \,, \end{aligned}$$

as the local time separation function $\tilde{\omega }$ of $\tilde{U}$ (with respect to $\tilde{X}$) is continuous and finite. Finally, $\lambda$ is inextendible as a geodesic in *X* since it is not even extendible as a continuous curve ($\lim _{t\nearrow t_*}\iota \circ \lambda (t) = \lim _{t\nearrow t_*}\gamma _{q,\tilde{p}}(t)= \tilde{r}\notin \iota (X)$)—thus contradicting property (*TC*). $\square$

We can now relate the low-regularity inextendibility to a blow-up of curvature. More precisely, we have the following result.

### Theorem 5.4

Let $(X,d,\ll ,\le ,\tau )$ be a strongly causal Lorentzian length space that has property (*TC*). If *X* is extendible, the extension has a causal curvature singularity [22, Def. 4.20]. Specifically, the extension cannot have bounded upper causal curvature.

### Proof

Let $(X,d,\ll ,\le ,\tau )$ be a Lorentzian length space that is strongly causal and has property (*TC*). Assume that there exists a Lorentzian length space $(\tilde{X},\tilde{d},\tilde{\ll },\tilde{\le },\tilde{\tau })$ extending $(X,d,\ll ,\le ,\tau )$ and having causal curvature bounded above. Then [22, Rem. 4.16, Thm. 4.17 and Thm. 4.18] yield that $(\tilde{X},\tilde{d},\tilde{\ll },\tilde{\le },\tilde{\tau })$ is regular. This contradicts the inextendibility result Theorem 5.3 and yields that *X* has a curvature singularity in the sense of [22, Def. 4.20]. $\square$

We now specialise to the case where the object to be extended is a smooth spacetime. Firstly, recall that *causally plain* spacetimes are precisely those that do not exhibit the bubbling phenomenon. Roughly speaking, a metric is bubbling if it contains a point where the boundary of the future null cone has non-empty interior. (For a precise definition, see [7, Definition 1.16]; cf. also the discussion preceding Lemma 5.6 in [22].) Spacetimes (*M*, *g*) with *g* a Lipschitz metric are causally plain [7, Corollary 1.17].

The following result is now a direct corollary of Theorem 5.3.

### Corollary 5.5

Let (*M*, *g*) be a smooth, strongly causal and timelike geodesically complete spacetime and let $(M,d^h,\ll ,\le ,\tau )$ be its induced Lorentzian length space. Then $(M,d^h,\ll ,\le ,\tau )$ is inextendible as a regular Lorentzian length space, and hence also inextendible in the class of continuous, strongly causal and causally plain spacetimes that are regular.

### Proof

By Lemma 5.2$(M,d^h,\ll ,\le ,\tau )$ has property (*TC*) and strong causality is the same notion for spacetimes and the corresponding Lorentzian length spaces by [22, Lemma 2.21(i),(ii) and Lemma 2.38(iii)]. Thus, Theorem 5.3 applies, showing that $(M,d^h,\ll ,\le ,\tau )$ is inextendible as a regular Lorentzian length space. Furthermore, by [22, Thm. 5.12] every continuous strongly causal and causally plain spacetime $(\tilde{M}, \tilde{g})$ gives rise to a Lorentzian length space. $\square$

Also in this case of spacetimes we obtain the result that timelike geodesic completeness forces the extension to have a curvature singularity, even though curvature cannot be defined in the usual sense via the Riemann tensor.

### Corollary 5.6

Let (*M*, *g*) be a smooth, strongly causal and timelike geodesically complete spacetime and let $(M,d^h,\ll ,\le ,\tau )$ be its induced Lorentzian length space. If $(M,d^h,\ll ,\le ,\tau )$ is extendible as a Lorentzian length space then, the extension has a causal curvature singularity. (It cannot have causal curvature bounded above.)

### Proof

This follows directly from Theorem 5.4, similarly to the proof of Corollary 5.5. $\square$

### Remark 5.7

In [1], Alexander and Bishop introduced sectional curvature bounds for general semi-Riemannian manifolds. Moreover, they characterised these curvature bounds via triangle comparison with small triangles in model spaces (i.e. the spaces $M_K$ from (1) in the Lorentzian setting), see [1, Thm. 1.1]. As was shown in [22, Ex. 4.9], our definitions in Sect. 2 are compatible with these curvature bounds in this sense and in particular a curvature singularity in our sense implies that there cannot be a corresponding sectional curvature bound in the sense of [1]. Corollary 5.6 therefore implies that if the extension is assumed to be a smooth and strongly causal spacetime itself, then its sectional curvature as defined in [1] must be unbounded above.

To conclude this section we note that it is an interesting open question whether one can characterise completeness of timelike geodesics in Lorentzian length spaces via condition (*TC*), analogous to the smooth case, cf. [35, p. 154].

## Relation to other results on low-regularity inextendibility

In this final section we relate our work to further current results on the low-regularity inextendibility of spacetimes.

In [18] it was recently established that in a (locally) Lipschitz continuous spacetime maximal causal curves have a causal character. This immediately gives that the induced Lorentzian length space $(M,d^h,\ll ,\le ,\tau )$ of a strongly causal Lipschitz spacetime (*M*, *g*) is regular: By [7, Cor. 1.17] and [22, Thm. 5.12] $(M,d^h,\ll ,\le ,\tau )$ is a Lorentzian length space and by [18, Thm. 1.1] it is regular (a fact that was already observed by Graf and Ling in [18]). From this they deduce that a timelike geodesically complete smooth spacetime is inextendible in the class of Lipschitz spacetimes. Thus, their result is slightly stronger than ours when restricted to spacetimes (compare Corollary 5.5) as they do not need strong causality of the original spacetime. However, even when restricting to the case where the object to be extended is a spacetime, our result is more general in the following sense:

- It allows the original spacetime to be of low regularity (continuous and causally plain) as well.
- There might be continuous strongly causal, causally plain spacetimes inducing a regular Lorentzian length space where the metric is not locally Lipschitz continuous.
- It applies even to non-manifold extensions, and
- It relates inextendibility with curvature blow-up (Theorem 5.4).

In [15] the authors show that a smooth, timelike geodesically complete and globally hyperbolic spacetime is $\mathcal {C}^0$-inextendible, i.e. there is no spacetime with continuous metric extending the given spacetime. Again, as above, their result is slightly stronger when restricting to spacetimes, since of course not all spacetimes with continuous metrics give rise to a Lorentzian length space, as they need not be causally plain and strongly causal (see, for example, [7, Ex. 1.11]). However, our approach does not need the original spacetime to be globally hyperbolic and (as above) allows it to be of low regularity as well. Moreover, as noted above our result also rules out non-manifold extensions (as long as they are regular Lorentzian length spaces). A closer inspection of the proof of Theorem 5.3 reveals that one does not need that the entire extension is regular. In fact, all that is needed is that a maximal causal curve $\gamma$ that is contained in the original space except for its endpoint (which is on the boundary) is timelike whenever its starting point and endpoint are timelike related in the extension. This is weaker than being regular, as it essentially only concerns points in the original space and its boundary. Thus, the main result of [15] can be understood in this way: If the smooth spacetime is timelike geodesically complete and globally hyperbolic, then maximal causal curves as above have a causal character. This then yields the inextendibility result.

It should also be noted that in our framework one can define *future/past one-connectedness* [38, Def. 2.13] and *future/past divergence* [14, Def. 2.4(2)] as for spacetimes. Since being extendible forces the future or past boundary to be non-empty by Lemma 3.8, a further line of study could be to see if, as for spacetimes, future (past) one-connectedness together with future (past) divergence yields empty future (past) boundary (cf. [14, Thm. 2.5]).

To summarise, we have developed a framework where we can show inextendibility of spaces that resemble timelike geodesically complete spacetimes, in a similar spirit as the classical result [3, Prop. 6.16]. Our approach provides a new and unified perspective on the recent results [15, 18], see the discussion above. Moreover, for the first time we can relate low-regularity inextendibility with a (synthetic) curvature blow-up—a fact that fits well with physical expectations. Finally, it shows that timelike geodesic completeness is a very robust property, which carries over even to spaces that are not spacetimes or even manifolds.

## Footnotes

1 That is, one implicitly must assume that the metric is smooth on $\iota (M)$ in the coordinate chart on $\tilde{M}$ in which the metric is just continuous.

2 See, for instance, [5] for an illustration of this example.
